# Association between multiple sclerosis and epilepsy: large population-based record-linkage studies

**DOI:** 10.1186/1471-2377-13-189

**Published:** 2013-12-04

**Authors:** Alexander N Allen, Olena O Seminog, Michael J Goldacre

**Affiliations:** 1 University of Oxford, Brasenose College, Radcliffe Square, Oxford OX1 4AJ, UK; 2 Unit of Health–Care Epidemiology, Nuffield Department of Population Health, University of Oxford, Old Road Campus, Old Road, Oxford OX3 7LF, UK

**Keywords:** Epilepsy, Multiple sclerosis, Risk, Coexistence, Record-linkage, Epidemiology

## Abstract

**Background:**

Multiple sclerosis (MS) and epilepsy are both fairly common and it follows that they may sometimes occur together in the same people by chance. We sought to determine whether hospitalisation for MS and hospitalisation for epilepsy occur together more often than expected by chance alone.

**Methods:**

We analysed two datasets of linked statistical hospital admission records covering the Oxford Record Linkage Study area (ORLS, 1963–1998) and all England (1999–2011). In each, we calculated the rate of occurrence of hospital admission for epilepsy in people after admission for MS, compared with equivalent rates in a control cohort, and expressed the results as a relative risk (RR).

**Results:**

The RR for hospital admission for epilepsy following an admission for MS was significantly high at 4.1 (95% confidence interval 3.1–5.3) in the ORLS and 3.3 (95% CI 3.1–3.4) in the all-England cohort. The RR for a first recorded admission for epilepsy 10 years and more after first recorded admission for MS was 4.7 (2.8–7.3) in ORLS and 3.9 (3.1–4.9) in the national cohort. The RR for the converse–MS following hospitalisation for epilepsy–was 2.5 (95% CI 1.7–3.5) in the ORLS and 1.9 (95% CI 1.8–2.1) in the English dataset.

**Conclusions:**

MS and epilepsy occur together more commonly than by chance. One possible explanation is that an MS lesion acts as a focus of an epileptic seizure; but other possibilities are discussed. Clinicians should be aware of the risk of epilepsy in people with MS. The findings may also suggest clues for researchers in developing hypotheses about underlying mechanisms for the two conditions.

## Background

Multiple sclerosis (MS) is one of the commonest serious neurological conditions, with a prevalence of 0.1%, and is caused by focal demyelination of the brain. Epilepsy is also a common chronic neurological condition, with a prevalence of approximately 0.5% of the population, and is caused by abnormal electrical activity in the brain [1]. It is well known that focal lesions, such as tumours, haemorrhages or abscesses, can act as a nidus for epileptic seizures but there is a paucity of information on whether the focal lesions caused by MS can cause epilepsy. Although there have been studies examining the link between these two conditions, [2-6] which show an increase in the prevalence of epilepsy following a diagnosis of MS, most have been small. Any association between the two conditions is not well understood or widely recognised.

This study used two large record-linked statistical datasets, one for the Oxford region of England (from 1963–1998) and one for the whole of England (from 1999–2011), to determine whether MS and epilepsy co-occur in the same individuals more commonly than expected by chance. We used both datasets to determine whether there was consistency between them in the findings.

## Methods

### Population and data

We used data from English national Hospital Episode Statistics (HES, 1999–February 2011), provided by the NHS Information Centre (IC), and English national death registration data provided by the Office for National Statistics. We also used data from the Oxford Record Linkage Study (ORLS) which spans 1963 to 1998. Both HES and the ORLS include brief statistical abstracts of all hospital admissions and day case care. In English National Health Service (NHS) terminology, a day case is a patient who is admitted to hospital but who does not stay overnight. Both datasets also include data from death registrations. In each dataset, the abstracts for each individual are linked together into a cumulative record of successive hospital admissions, and death if it occurred, for each person. The population covered by the ORLS expanded from 850,000 people in years preceding 1974 to 1.9 million from 1975–1988, and 2.5 million people from 1989 onwards. The population of England is 55 million. The building of the linked datasets was done by staff of the Oxford Record Linkage Study at the Unit of Health-Care Epidemiology, University of Oxford. All data were anonymised by encryption of identifiers; and the encrypted identifiers were used to link successive records for the same individual.

#### Analysis

The same analysis was done for each of the diseases in combination as described below for MS preceding epilepsy. In each dataset, a cohort of people with a hospital admission or record of day case care for MS (which we termed the ‘exposure cohort’) was constructed by identifying the first admission, or episode of day case care, for MS as a reason for hospital care (in any diagnostic position on the hospital record) in an NHS hospital during the study period. The International Classification of Diseases (ICD) code used for MS was G35 in the tenth revision of the International Classification of Diseases, with equivalent codes in ICD 7, 8 and 9 in the ORLS. A second cohort, to be used as a ‘control cohort’, was constructed by identifying the first admission for each individual with various other, mainly minor medical and surgical conditions (see Table footnotes), as used by us in other studies of associations between diseases [7,8]. In this design, the standard epidemiological practice was followed, when hospital controls are used, of using a broad range of conditions, rather than relying on a narrow range (in case the latter are themselves atypical in their risk of subsequent disease). People were excluded from the MS or control cohort if they had an admission for epilepsy either before or at the same time as the admission for MS or the control condition.

We then searched the linked database for any subsequent record of epilepsy as the ‘outcome’ condition. We used the ICD10 codes G40–G41 for epilepsy and their ICD 7, 8 and 9 equivalents.

#### Statistical methods

We calculated rates of epilepsy using person-years. We took ‘date of entry’ into each cohort – MS and control cohort – as the date of first admission for MS, or control condition, and ‘date of exit’ as the date of first record of subsequent epilepsy, death, or the end of study (December 31 1998 for ORLS, February 28 2011 for England), whichever was earliest. We calculated rates for epilepsy, stratified and then standardised by age (in five-year age groups), sex, calendar year of first recorded admission, region of residence, and quintile of patients’ Index of Deprivation score (as a measure of socio-economic status, only available for the all-England dataset). We standardised by using the combined MS and control cohorts as the standard population. The stratum-specific rates of epilepsy in the standard population were applied to the number of people in each stratum in the MS cohort, separately, and then to those in the control cohort, to give an observed (O) and expected (E) number of people with epilepsy in each of the two cohorts. The relative risk of epilepsy in the MS cohort, relative to that in the control cohort, was calculated using the formula (O^MS^/E^MS^)/(O^contr^/E^contr^), where O is the observed and E the expected number of cases of epilepsy in the MS and control cohorts. These calculations, and the calculation for the confidence interval for the relative risk and χ^2^ statistics for its significance, were undertaken using standard published statistical methods [9].

We then reversed the procedures and repeated the analyses for epilepsy as the ‘exposure’ condition and MS as the ‘outcome’. We excluded anyone who had an admission for MS prior to the first record of epilepsy. These methods provided results without double-counting any individual (i.e. no individual appears in both the MS-epilepsy and the epilepsy-MS analyses).

The datasets are in the custodianship of the Unit of Health-Care Epidemiology and are not freely available. Ethical approval to construct, maintain, develop and analyse the datasets has been obtained, on an ongoing basis, from the Central and South Bristol Multi–Centre Research Ethics Committee (04/Q2006/176).

## Results

There were 3 913 people in the MS cohort in the ORLS dataset and 85 772 in the all-England dataset. There were 18 790 people in the epilepsy cohort in the ORLS data and 520 203 in the all-England data. Table 1 shows the age distribution in each cohort, in each dataset, and the percentages in each age group who were female.

**Table 1:** Number (N) and percentage of people with multiple sclerosis or epilepsy in each age group, and percentage of women in each age group (in parentheses)

**Age at admission for the exposure condition (years)**	**Multiple sclerosis**		**Epilepsy**	
	**ORLS, N=3 913, (% of women)**	**England, N=85 772, (% of women)**	**ORLS, N=18 790, (% of women)**	**England, N=520 203, (% of women)**
<15	0.3 (60.0)	0.1 (58.1)	23.3 (43.7)	8.8 (45)
15–34	26.7 (70.1)	12.4 (71.0)	24.2 (47.5)	19.3 (49)
35–54	45.2 (65.8)	45.4 (70.2)	18.7 (44.3)	25.6 (46)
5–64	15.4 (62.4)	21.6 (66.1)	9.6 (39.3)	12.9 (46)
65+	12.4 (66.7)	20.5 (69.0)	24.3 (49.7)	33.4 (53)
Total	100.0 (66.5)	100 (69.2)	100.0 (45.8)	100 (49)

### Admission for MS followed by admission for epilepsy

Following hospital admission for MS, there was a significantly high risk of subsequent hospital admission for epilepsy in both datasets (Table 2). The relative risk, comparing the MS cohort with the control cohort, was 4.1 (95% confidence interval 3.14–5.27) in the ORLS and 3.3 (3.14–3.40) in the all-England cohort (Table 2).

**Table 2:** **Occurrence of epilepsy in people admitted with multiple sclerosis, and of multiple sclerosis in people admitted with epilepsy: observed (O) and expected number (E) of cases in each cohort, relative risk (RR)**^**a **^**in the exposure cohort compared with the control cohort**^**b**^**, with its 95% confidence intervals (95% CI), and *p *** **values, by time intervals between the two conditions**

**Exposure cohort**	**Time interval**	**Outcome**	**ORLS (1963–1998)**				**England (1999–2011)**			
			**O**	**E**	**RR (95% CI)**	***p *** **value**	**O**	**E**	**RR (95% CI)**	***p *** **value**
Multiple sclerosis	<1 year	Epilepsy	13	3.6	3.6 (1.92–6.23)	<0.001	574	370.0	1.6 (1.44–1.70)	<0.001
	1–4 years		16	4.3	3.7 (2.12–6.08)	<0.001	1194	251.0	4.9 (4.66–5.24)	<0.001
	5–9 years		14	3.2	4.4 (2.41–7.48)	<0.001	726	166.0	4.5 (4.20–4.88)	<0.001
	10+ years		19	4.1	4.7 (2.80–7.34)	<0.001	86	22.8	3.9 (3.11–4.85)	<0.001
	Total		62	15.3	4.1 (3.14–5.27)	<0.001	2580	809.7	3.3 (3.14–3.40)	<0.001
Epilepsy	<1 year	Multiple sclerosis	9	3.3	2.9 (1.29–5.67)	0.003	206	196.1	1.1 (0.91–1.21)	0.48
	1–4 years		9	3.4	2.7 (1.21–5.23)	0.006	394	154.0	2.8 (2.52–3.13)	<0.001
	5–9 years		6	2.7	2.3 (0.82–5.09)	0.083	206	90.5	2.4 (2.10–2.83)	<0.001
	10+ years		9	4.2	2.2 (0.99–4.24)	0.034	19	9.9	2.0 (1.19–3.27)	<0.005
	Total		33	13.6	2.5 (1.71–3.54)	0.001	825	450.4	1.9 (1.79–2.07)	<0.001

^a^All analyses were undertaken initially within five-year age groups (and within the other strata used), using the combined MS and control cohorts (or the combined epilepsy and control cohorts) as the ‘standard’, to provide observed and expected numbers within (1) the MS cohort (or epilepsy cohort) and (2) the control cohort, within each stratum group. The stratum-specific observed and expected numbers in each cohort were then summed to give an age-standardised all-ages total, adjusted for age in 5-year bands, time period in single calendar years, region of residence and deprivation score associated with patients’ area of residence, in quintiles.

^b^Conditions used in control cohort, with Office of Population, Censuses and Surveys (OPCS) code edition 4 for operations and ICD10 code for diagnosis (with equivalent codes used for other coding editions): adenoidectomy (OPCS4 E20), tonsillectomy (F34, F36), appendectomy (H01–H03), dilation and curettage (Q10–Q11), hip replacement (W37–W39), knee replacement (W40–W42), squint (ICD10 H49–H51), cataract (H25), otitis (H60–H67), varicose veins (I83), haemorrhoids (I84), upper respiratory tract infections (J00–J06), deflected septum, nasal polyp (J33+J34.2), impacted tooth and other disorders of teeth (K00–K03), inguinal hernia (K40), in-growing nail, toenail and other diseases of nail (L60), bunion (M20.1), internal derangement of knee (M23), contraceptive management (Z30).

### Admission for epilepsy followed by admission for MS

In the cohort of people with epilepsy, similarly, there was an increased risk of MS: 2.5 (1.71–3.54) in the ORLS and 1.9 (1.79–2.07) in the England cohort (Table 2). The relative risks were higher in the ‘MS followed by epilepsy’ analysis than in the ‘epilepsy followed by MS’ analysis (Table 2).

### Time intervals and age-groups

Table 2 shows analyses subdividing the associations by time interval between first recorded admission for MS and first recorded admission for epilepsy. Relative risks were similar at each time interval, whether short or long. The risk was highest in the younger age groups (those under 55 years, Table 3).

**Table 3:** **Occurrence of epilepsy in people admitted with multiple sclerosis, and of multiple sclerosis in people admitted with epilepsy: observed (O) and expected number (E) of cases in each cohort, relative risk (RR)**^**a **^**in the exposure cohort compared with the control cohort**^**b**^**, with its 95% confidence intervals (95% CI), and *p *** **values, by age group**

**Exposure disease**	**Age at admission for exposure disease (years)**		**ORLS (1963–1998)**				**England (1999–2011)**			
		**Outcome**	**O**	**E**	**RR (95% CI)**	***p *** **value**	**O**	**E**	**RR (95% CI)**	***p value***
Multiple sclerosis	<35	Epilepsy	30	3.8	8.0 (5.34–11.39)	<0.001	358	99.3	3.7 (3.28–4.05)	<0.001
	35–44		15	3.6	4.2 (2.35–7.07)	<0.001	587	159.0	3.9 (3.59–4.26)	<0.001
	45–54		11	3.3	3.4 (1.66–6.11)	<0.001	780	237.0	3.6 (3.34–3.87)	<0.001
	55–64		4	1.9	2.1 (0.57–5.46)	0.247	508	173.0	3.1 (2.81–3.37)	<0.001
	65–74		1	2.0	0.5 (0.01–2.81)	0.728	253	98.2	2.6 (2.31–2.98)	<0.001
	75+		1	0.6	1.7 (0.04–9.34)	0.894	94	52.0	1.8 (1.46–2.22)	<0.001
	Total		62	15.3	4.1 (3.14–5.27)	<0.001	2580	809.7	3.3 (3.14–3.40)	<0.001
Epilepsy	<35	Multiple sclerosis	10	6.3	1.6 (0.76–2.98)	0.203	176	79.6	2.4 (2.01–2.77)	<0.001
	35–44		8	2.6	3.2 (1.37–6.52)	0.002	202	96.2	2.2 (1.92–2.59)	<0.001
	45–54		9	2.3	4.2 (1.87–8.25)	<0.001	214	111	2.1 (1.77–2.36)	<0.001
	55–64		3	1.8	1.7 (0.35–5.32)	0.569	110	81.3	1.4 (1.13–1.68)	<0.001
	65–74		3	0.5	6.8 (1.23–24.57)	0.006	83	48.2	1.8 (1.41–2.23)	<0.001
	75+		0	0			40	33.6	1.2 (0.85–1.66)	0.298
	Total		33	13.6	2.5 (1.71–3.54)	<0.001	825	450.4	1.9 (1.79–2.07)	<0.001

^a, b^see footnotes Table 2.

Analyses were also done separately for males and females, and for records in which MS and epilepsy were the main diagnosis (rather than recorded in any part of the hospital admission record). Relative risks in these subsets were similar to those presented here.

### Absolute risk approximations

Because we do not have data on out-migration, we cannot give precise estimates of absolute risk. However, using the English population (in which out-migration is less of an issue than with the Oxford population), we can give an approximation. In the English cohort of 85 772 people with MS (Table 1), there were 2 580 who had an admission for epilepsy (Table 2). This gives an approximate absolute risk of epilepsy of 3% in people with MS.

## Discussion

### Main findings

In these large population-based studies we confirm that there is a statistically significant association between MS and epilepsy. The elevation of risk of epilepsy after MS was 3- to 4-fold. It was highest in the youngest age groups. The study also shows an association in the opposite direction, though less strong, between admission for epilepsy followed by a subsequent admission for MS.

### Comparisons with existing literature

Previous studies have reported that the risk of epilepsy after MS is between three to six times higher than that in people without MS [2-6]. One study showed that the peak increase in epilepsy occurred between four and seven years after the diagnosis of MS [10]. A review of 29 studies (covering a total of 389 patients with MS and epilepsy), published in 2003, concluded that there is evidence for an association between epilepsy and multiple sclerosis but suggested that the association may not be one of MS causing epilepsy [11].

Our study covered a much larger population than previous studies (it reports on 2642 patients with a record of epilepsy after MS, and 858 with an admission for MS after an admission for epilepsy).

Our estimates of the absolute risk of 3.0% for epilepsy in people with MS are slight underestimates, as described in the Results, but are similar to findings of 3.5% previously reported [12].

### Possible mechanisms and implications

While an explanation of mechanisms behind these associations is beyond the scope of this study, the literature provides suggestions about some potential mechanisms. Lebrun showed that the frequency of seizures correlates with the number of flare-ups of MS [13]. Two papers by Calabrese et al. report that patients with MS and epilepsy show more severe cortical inflammation, and a higher number of intracortical lesions, than those with MS alone [14,15]. Waxman put forward the hypothesis that the abnormal sodium channel expression found in the neurones of some patients with MS could play a role both in the initial development of MS and in the subsequent occurrence of epilepsy [16]. These sodium channel abnormalities, if they preceded the demyelinating changes of MS, could also explain epilepsy preceding MS.

If an MS lesion acts as a focus of an epileptic seizure, it might be expected that there would be a correlation between the location of the lesion and the origin of the seizure. However, as the datasets do not include a record of the location of lesions, and do not record the type of seizure, a study would have to be specifically set up to test this hypothesis.

The weaker relationship between prior epilepsy and subsequent MS, as found in this study, has several possible explanations. First, because the study is based on hospital admissions, it is possible that some people will have had clinical MS, and some will have had epilepsy, without being admitted to hospital; and others will have had admissions for either before the start of the datasets. Thus, we cannot be sure about the sequence of the temporal relationships. Nonetheless, it seems unlikely that the high relative risks for epilepsy in people first admitted 10 years and more after an admission for MS – 4.1 in the ORLS, 3.9 in England (Table 2) – could be wholly explained by epilepsy that actually preceded MS.

It is also possible that, in a small proportion of patients subsequently diagnosed with MS, seizures were the first symptom of MS; one paper reports that seizures were the initial clinical manifestation of MS in 10% of MS patients [17].

It is also possible that treatment of MS, or of epilepsy, may affect the association between the two diseases; but we had no treatment data to test this. Results of clinical trials on management of MS with interferon were published in early 1990s and we assume that this treatment became more widely prescribed from the mid-1990s onwards. It is worth noting that the RRs in the ORLS (largely pre-interferon) and the all-England dataset (post-interferon) were similar. Finally, there could be underlying contributing factors that, independently, increase a person’s risk of developing both MS and epilepsy, which would be an interesting area for future research.

The findings could have important implications for clinicians, first, in needing to be aware of the risk of epilepsy in people with MS, and, second, because status epilepticus may cause sudden death. Clinicians may wish to judge whether individual patients with MS, and their relatives, should be told about epilepsy risk or whether to spare them concerns about it. On the one hand the risk is uncommon, although at 3% it is not rare either. On the other, the clinician might wish to warn and educate the patient with MS, and his/her family, about what to do in the event of a seizure. The clinician may particularly want to consider whether the patient has any other risk factors for epilepsy, such as medication and alcohol use, that would potentially exacerbate this risk.

### Strengths and weaknesses

The study is large and was undertaken in geographically defined populations. The Oxford dataset covers just a regional population but includes long duration of follow-up, whereas the English dataset is much larger in size but with shorter follow-up periods. There are some weaknesses in this study. It is confined to people who received day case care or hospital admission, both for the identification of MS and for epilepsy. Data are not recorded on people who have left the area covered by the ORLS dataset (or those who leave England), and it has been assumed that the rates of outward migration would be similar in people with MS, epilepsy, and the control population. These are reasons for using a ‘control’ cohort from within the datasets. We did not have data on the criteria used to diagnose either epilepsy or MS, or on the type of seizures. We relied wholly on the available ICD codes. Current privacy regulations in England preclude the possibility of sampling records and checking codes against diagnostic information in patients’ original case records. However, the fact that the findings from the present very large study (e.g. the absolute risk of 3%) are similar to the findings from much smaller studies based on clinical diagnostic criteria [2,4,13] indicates that our findings are likely to be reliable.

Changes in diagnostic imaging, in particular the increased use of MRI, may have resulted in more cases of MS being diagnosed in recent years, although it can be assumed that this would affect both the study cohorts and the control cohort equally. It is noteworthy that both datasets – covering different time periods in which there will have been different regimes of investigation and treatment gave similar results. As discussed above, we cannot be sure about the temporal sequence of MS and epilepsy. It would need a study of patients’ life-long full medical record to determine which disease came first.

We did a sub-study of time intervals between MS and epilepsy, and vice versa, to consider the possibility of surveillance bias. This bias can occur when the fact of being diagnosed with one condition increases the chance that another condition is found, investigated and recorded in the patient’s case record. If there were surveillance bias, a relative risk would be expected that is highest at short time intervals after the first diagnosis, and which declines as the interval increases. This was not found: this indicates that surveillance bias has had only a minimal role, if any, and that, for some people, there may be a long latency period between the onset of MS and the manifestation of epilepsy.

## Conclusions

This study is the largest of its kind so far and provides strong evidence for a link, possibly bidirectional, between MS and epilepsy. This could have important implications for clinicians in needing to be aware of the risk of epilepsy in people with MS. It also has implications for researchers in developing and testing hypotheses about underlying mechanisms for the two conditions.

## Competing interests

None of the authors has any conflict of interest to disclose.

We confirm that we have read the Journal’s position on issues involved in ethical publication and affirm that this report is consistent with those guidelines.

## Authors’ contributions

AA conceived of the study, conducted the literature review and wrote the manuscript. OS conducted the statistical analysis and reviewed and edited the manuscript. MG oversaw the design of the study and considerably edited the manuscript. All authors read and approved the manuscript.

## Pre-publication history

The pre-publication history for this paper can be accessed here:

http://www.biomedcentral.com/1471-2377/13/189/prepub
